# Supplementary material for: Are carbon emissions trading and green financial instruments synergistic? -Comprehensive quantitative research based on content analysis
Source: PLoS One. 2024 Mar 7;19(3):e0298601. doi: 10.1371/journal.pone.0298601 (PMC10919601; doi:10.1371/journal.pone.0298601)
Supplement: S1 Data — (ZIP) [file pone.0298601.s001.zip › Data/地级市Z_F工作报告.zip!/╜¡╦╒/─╧╛⌐╩╨/└┤╫╘╣1⁄2╓┌║┼í╛┬φ┐╦╩2╛▌═°í┐_2013.docx]

各位代表：

　　现在，我代表南京市人民政府，向大会报告工作，请予审议，并请政协委员和其他列席人员提出意见。

　　一、本届政府工作回顾

　　2012年，面对宏观经济形势复杂多变、经济下行压力不断增大的困难挑战，全市人民在省委省政府及市委的正确领导下，紧紧围绕率先基本实现现代化总目标，团结奋斗，攻坚克难，全力以赴稳增长、调结构、促转型、惠民生，使经济社会继续保持稳定健康发展。预计全年实现地区生产总值7200亿元，按可比价计算比上年增长12%左右；公共财政预算收入733亿元，增长15.4%；工业增加值2740亿元，增长11.3%；第三产业增加值3845亿元，增长12.1%；完成全社会固定资产投资4650亿元，增长16%；实现社会消费品零售总额3150亿元左右，增长17%；实际到账注册外资41亿美元以上，增长18%；万元地区生产总值能耗降低6.5%左右；城市居民人均可支配收入36700元，增长14%，农民人均纯收入15000元左右，增长14.4%；居民消费价格涨幅控制在3%以内。除因国际市场变化导致出口未能达到预定增幅目标外，基本完成十四届人大五次会议提出的各项目标。至此，本届政府已圆满完成了市人大各次会议确定的主要任务。

　　五年来，本届政府高举中国特色社会主义伟大旗帜，坚持以邓小平理论、“三个代表”重要思想和科学发展观为指导，开拓创新，务实奋进，率先建成了惠及全市人民的小康社会，开启了率先基本实现现代化的新征程。

　　这五年，综合实力显著增强。地区生产总值从2007年的3340亿元跃上7000亿元台阶，近三年年均新增1000亿元以上，五年翻了一番。公共财政预算收入从2007年的330亿元跃上700亿元台阶，近三年每年新增近100亿元，五年翻了一番以上；全社会固定资产投资是2007年的2.5倍；规模以上工业总产值为11400亿元，五年翻了近一番；三次产业增加值比例由2007年的3.5：48.1：48.4，调整为2012年的2.6：44.0：53.4；社会消费品零售总额年均增长17.3%；进出口总额年均增长8.6%；五年累计实际到账注册外资达153亿美元，新增私营企业注册资本2203亿元，新增个体工商户注册资金219亿元。结构调整、产业转型成效显著，实施“千企升级”计划、科技创新创业计划、新兴产业发展计划、现代服务业发展计划等，提升电子信息等四大支柱产业发展水平，推进智能电网、新型显示、生物医药等八大新兴产业，以及软件和信息服务、金融保险等八大现代服务业发展。经过近三年的治理，目前燕子矶地区66家化工企业已全部停产，335家“三高两低”企业得到整治，南京主城突破了化工围城、异味扰民的“环境困局”。首家被国家工信部授予中国软件名城称号，软件业务收入超过2000亿元。出台鼓励科技创新创业20条、"1+8"系列政策、“科技九条”等，推进“紫金科技创业特别社区”、软件谷、麒麟生态科技园、模范马路创新街区等载体建设。实施“紫金人才”和“321人才”计划，入选国家“千人计划”人才116名。研发经费支出占地区生产总值比重较上年提高0.2个百分点，专利授权量达1.65万件，是2007年的4.36倍。研究出台鼓励扩大内需的政策措施。综合保税区获国务院批准设立，江宁开发区升格为国家级开发区，金陵海关获批挂牌。承办了第四届世界城市论坛、中欧领导人会晤和中欧工商峰会、海峡两岸紫金山峰会、台湾名品交易会等重大国际国内展会。

　　这五年，民生保障得到显著改善。城乡公共服务支出占公共财政比例从2007年的61.4%提高到2012年的70%。制定实施城乡居民收入“双倍增计划”，城市居民人均可支配收入年均增长12.5%，农民人均纯收入年均增长13.3%；连续三年出台关于改善民生的“一号文件”，完善了10项民生政策，重点解决了10类特殊群体的困难；市区两级财政分期投入139亿元，解决了1983年以来近52万被征地人员进城保问题，为他们解除了后顾之忧，使他们成为真正的市民，生活得更有尊严；投入500多亿元，实施了南京历史上最大的安居工程，四大保障房组团1000多万平方米全面建设，首批住户顺利入住，改善了市民住房条件；2011年出台了全国最早的学前教育惠民举措，约有17万左右适龄儿童每人每年获得2000元助学券；医药卫生体制改革深入推进，基本医疗、公共卫生服务和医疗保障实现全覆盖，基本药物制度全面实施，减少了城乡居民医疗支出；千方百计扩大就业，五年新增城镇就业98万人；扩大社会保险覆盖面，五项险种累计参保人数达1296万人次；实施70岁以上老人和残疾人免费乘车、60至69岁老人半价乘车等惠民政策，开展了残疾人“助学、助听、助行、助业”等四助活动。

　　这五年，公共服务水平不断提高。统筹历史文脉传承和文化事业、文化产业发展。孙权纪念馆、非物质文化遗产博物馆、金陵图书馆、妇女儿童活动中心建成使用，六朝博物馆、江宁织造府、大报恩寺遗址公园、南京直立猿人化石遗址公园等文化项目加速推进。公共文化服务体系不断完善，基层文化服务设施全面提档升级，文化产业增加值达330亿元，年均增长25%以上。鼓楼医院、妇幼保健院、儿童医院等一批医疗机构改扩建工程竣工，河西医疗中心等工程加快建设。平安南京建设不断推进，社会整体发展稳定有序，群众的安全感进一步增强。畅通人民信访渠道，社区建设和管理得到加强，和谐社会建设不断推进。玄武湖和中山陵陵寝两大核心景区免费对市民开放。对外交流、宁台合作、涉外侨务、民族宗教工作不断拓展，友好城市和友好合作城市达47个。佛顶骨舍利盛世重光。荣获全国双拥模范城市“七连冠”。成功申办2014年青年奥林匹克运动会和2013年亚洲青年运动会。顺利举办历史文化名城博览会和中国航海日活动。先后荣获联合国人居奖特别荣誉奖、全国民族进步模范集体、联合国国际花园城市金奖、全国文明城市等光荣称号。

　　这五年，城市功能品质明显提升。围绕建设现代化国际性人文绿都目标和“一城三区”总体规划布局，修编了新一轮城市总体规划，实施了“三个提升”计划和河西新城等十大功能板块建设。南京长江隧道、长江四桥、绕越高速、玄武大道快速化改造等一批引导支撑城市发展的重大交通项目相继建成。纬一路和绕城公路改造加快推进，纬三路过江隧道建设、城西片区快速路网改造进展顺利。京沪高铁、沪宁城际铁路及南京南站建成运营，宁杭和宁安城际铁路、禄口机场二期工程、红花机场迁建等项目有序推进。地铁1号线南延和2号线竣工运营，开工建设地铁3号线、10号线一期、4号线一期、宁高一期等轨道交通线。河西新城青奥村和一批功能性大楼建设进展顺利。老城南文化街区保护、下关滨江旧区改造、燕子矶化工区整治、浦口新城和麒麟生态科技园开发等逐步推进。实施国家生态市、国家生态园林城市、国家森林城市“三城”同创，推进大气环境、水环境、垃圾分类处理、节能减排、“动迁拆违、治乱整破”等专项行动。基本完成玄武湖、金川河“一湖一河”区域雨污分流和城北护城河、外金川河环境综合整治任务。五年累计完成小区出新271个，整治街巷1509条。明外郭-秦淮新河百里风光带、滨江两岸百里风光带和紫金山-玄武湖中央公园及百个小游园等建设全面启动，人均公园绿地面积达14.2平方米。

　　这五年，城乡统筹一体发展逐步加快。大力推进“区区县县通轻轨、干线公路连街镇、区域供水全覆盖、城乡公交一体化、水利能力大提升”和郊区县先进制造业基地建设，促进城乡规划、产业发展、要素配置、基础设施和公共服务一体化。深入推进跨江发展，江北地区发展明显加快。一批好的先进制造业和新兴产业项目落户郊区县，郊区县地区生产总值占全市的比重达54%。以农业“1115工程”为抓手，推动现代农业发展和国土资源保护，粮食生产连续九年获得丰收，发展乡村旅游、生态旅游。江北大道、122省道、宁高新通道三条城乡大动脉和340省道、247省道等干线公路建设全面实施。堤顶宽8米的长江干堤提升工程完成73公里，滁河和水阳江治理加速推进，大大提高了防洪能力和生态水平。基本完成4482个村庄环境整治任务。加大了城乡帮促力度，市区县为204个集体经济薄弱村建造100多万平方米标准厂房，基本消除了可支配收入低于50万元的行政村。

　　这五年，服务型政府建设进一步加强。实行了“大部制”改革，推进了审批制度、简政强区县、国资管理、财政体制、土地利用、城市管理等综合改革，向区县和开发区下放了202项市级行政权力。健全了重大行政决策咨询、论证、听证等制度。建成市级行政服务中心，开通运行"12345"政府服务呼叫中心。制定并实施了全面推进依法行政五年规划，制定和修订政府规章24部，向市人大常委会提交立法议案29部。实施项目联审联评，推进部门公开办事程序、时限等服务承诺—展“联系市民、服务百姓”、“改进服务、千企走访”和“向人民汇报”民主评议直播等活动，为人民群众生活和企业发展排忧解难。与此同时，人防、司法、工会、妇女儿童、慈善、档案、地方志、社会科学等各项工作取得新的进步。

　　过去的五年，我们实现了全面小康的奋斗目标，为率先基本实现现代化夯实了基础，积累了经验。我们坚持科学发展不动摇，不断提升发展标杆，推动经济社会转型创新跨越发展，使整体实力、发展水平不断跃上新台阶；我们坚持以人为本、执政为民不动摇，不断满足人民群众的需求，解决人民群众关注的现实问题，让老百姓得实惠，使人民生活水平、社会保障水平不断跃上新台阶；我们坚持提升城市功能品质不动摇，集中力量推进一批事关全局、事关长远、事关民生的基础设施、功能设施、生态环境及文化项目建设，使城市承载能力、竞争力不断跃上新台阶；我们坚持全域统筹发展不动摇，构建城乡统筹、跨江发展、区域协调的新格局，使城乡面貌、公共服务、产业集聚不断跃上新台阶；我们坚持改革开放创新不动摇，沉着应对国际金融危机，着力解决发展中的体制机制障碍，维护社会公平正义，使经济发展活力、社会创新能力不断跃上新台阶。

　　各位代表，过去五年取得的辉煌成就，靠的是中国特色社会主义旗帜的正确指引，靠的是党中央国务院、省委省政府和市委的正确领导，靠的是全市广大人民群众的团结奋斗、顽强拼博和社会各界的鼎力支持。在此，我代表南京市人民政府，向在各行各业辛勤劳动的全市人民，向所有支持和关心南京建设和发展的朋友们、同志们，表示衷心的感谢！

　　与此同时，我们也清醒地看到，南京经济社会发展中还存在不少矛盾和困难：做大经济总量和产业结构调整转型升级、构建现代产业体系、转变发展方式的任务十分繁重；城乡居民收入、环境质量、郊县发展仍是我们需要主攻提升的“三大短板”；企业生产经营成本上升和创新能力不足的问题并存；公共服务水平滞后于经济发展，交通拥堵等压力亟需解决；各种体制机制显现出来的深层次矛盾有待化解。这些问题和矛盾，我们将在新一届政府工作中不断加以克服。

　　二、今后五年的发展目标

　　今后五年，是南京全面贯彻落实十八大战略部署、率先基本实现现代化的重要时期，是以“办亚青、办青奥”为契机提升城市竞争力的重大机遇期，是实施“十二五”规划“建设新南京、创造新生活”的关键时期。

　　从国内外形势来看，机遇和挑战并存，机遇大于挑战。我们面临着世界经济总体向好，发展空间不断拓展的机遇；面临着新一代数字化技术应用带来的生产方式、商业模式、生活方式变革，产业向高端发展的机遇；面临着扩大内需、提高创新能力、促进经济发展方式转变的机遇；面临着国家在关键领域推进改革、释放改革“红利”的机遇；面临着城乡统筹、新一轮城镇化发展的机遇。从南京来看，随着我市“十二五”规划的实施，以及一系列推动科学发展的政策举措逐步落实到位，全市经济社会必将保持平稳较快可持续发展，我们对南京的发展前景充满信心。

　　各位代表，我们深知，人民群众对南京的未来充满着期盼。期盼我们保持经济持续增长，做强整体实力，实现富民强市，为不断改善全市人民生活奠定物质基础，提供更多更好的就业创业机会，让人民群众收入更高、财富更多；期盼我们完善城市功能，提升承载能力，让市民出行更便捷，生活更便利；期盼我们建立包容、均等、优质的公共服务体系，为百姓提供更多更好的教育、医疗、住房保障等社会公共服务，让困难群众得到更多的帮扶；期盼我们下大功夫解决大气环境、水环境等生态文明建设中的突出问题，让天更蓝、水更清、空气更清新、城市更宜居。这些期盼，既是广大人民群众对美好生活的向往，也是政府工作的目标和重点，我们将为之坚持不懈地努力。

　　今后五年，我们要全面贯彻落实党的十八大和市十三次党代会精神，围绕“办青奥盛会、创率先大业、建人文绿都”的目标，坚持“创新驱动、内生增长、绿色发展”的道路，坚持“民生为先、统筹为要、生态为基、文化为魂”的方针，全面推进政治建设、经济建设、文化建设、社会建设、生态文明建设，着力打造“三都市、三名城”，逐步使美好的蓝图变为现实，使人民群众的期盼变为现实。

　　综合实力新跨越。牢牢把握科学发展这个主题，继续做大做强经济总量，确保2015年地区生产总值突破万亿，公共财政预算收入突破千亿，确保“十二五”末达到省定基本实现现代化目标，城市综合实力和发展能级显著提升。

　　产业升级新突破。以科技创新、结构调整为抓手，推动经济发展方式转变，使新兴产业成为支柱产业，传统支柱产业提档升级，创新创业潜力得到有效释放，真正把科技创新优势转化为产业优势、竞争优势，基本建成现代产业体系。

　　城市品质新提升。以办青奥为契机，加强城市基础设施建设，优化城市空间，完善城市功能，提升城市品质，保护古都神韵，延续历史文明，推进旧区更新，改善城乡面貌，城市国际化功能不断提升。

　　生态文明新形象。树立尊重自然、顺应自然、保护自然的生态理念，基本建成国家生态市、国家生态园林城市、国家森林城市，建设美丽乡村，充分彰显绿色、宜居特色，城乡生态环境质量明显改善。

　　人民生活新改善。大力实施城乡居民收入“双倍增计划”，想方设法提高人民群众收入，社会保障实现全覆盖，城乡居民充分就业，创业活力进一步增强，收入分配更趋合理，困难群众生活得到明显改善，人民生活更加幸福。

　　公共服务新水平。文化、教育、医疗、体育等社会事业全面进步，人民群众的基本权益得到有效保障，人口素质不断提高，社会公平正义得到切实维护，人民群众能享受到更多更好的覆盖城乡、功能完善、均等优质的公共服务。

　　城乡统筹新格局。加快推进新型城市化进程，基本建成郊区县先进制造业基地，“区区县县通轻轨、干线公路连街镇、区域供水全覆盖、城乡公交一体化、水利能力大提升”的蓝图基本实现，使现代农业大发展、农村环境面貌大变化、农村文明大提升。

　　行政能力新提高。践行以人为本、执政为民的理念，恪守为民、务实、清廉的准则，坚持依法行政、依法管理、依法办事，切实转变政府职能，做到干部清正、政府清廉、政治清明，初步建成学习型、法治型、服务型政府。

　　我们将围绕这些愿景，制订周详的规划、政策和具体的措施，积极整合资源，合理配置人力财力，努力让人民群众共享发展成果。

　　三、2013年经济社会发展主要任务

　　2013年是全面贯彻落实十八大战略部署的开局之年，是率先基本实现现代化和实施“十二五”规划的关键之年，是“办亚青、迎青奥”的落实之年，更是新一届政府履行职责的第一年，政府工作的总体要求是：全面贯彻落实党的十八大精神，以科学发展为主题，以加快转变经济发展方式为主线，以提高经济增长质量和效益为中心，以“稳中求进、以进促稳”为主基调，突出经济平稳较快增长，突出创新驱动结构调整转型升级，突出民生改善管理创新，突出城市功能建设品质提升，突出城乡统筹区域协调，突出环境保护生态建设，突出关键领域改革突破，集中力量主攻薄弱指标提升、城市面貌整体提升和办好亚青会，夺取率先基本实现现代化的阶段性胜利。

　　按照省委、省政府的部署和市委十三届四次全会的安排，2013年经济社会发展主要指标是：地区生产总值增长11%；公共财政预算收入增长13%；服务业增加值占地区生产总值比重提高1个百分点；全社会固定资产投资增长15%；社会消费品零售总额增长16%；实际利用外资增长14%；外贸出口增长5%；全社会研发经费支出占地区生产总值比重提高0.3个百分点；万元地区生产总值能耗降低7%以上、化学需氧量排放强度控制在1.55千克以内、二氧化硫排放强度控制在1.8千克以内；城镇居民人均可支配收入增长13%，农民人均可支配收入增长14%；居民消费价格指数控制在省定标准。

　　实现今年经济社会发展的目标，着重做好以下八个方面的重点工作：

　　（一）着力推动经济在结构调整转型升级中稳健增长，增强经济整体实力

　　坚持先进制造业、现代服务业“双轮”驱动，进一步优化生产力布局，积极推动产业向高端、高质、高效发展。

　　积极发展高端制造业。加速培育发展新一代信息技术、智能电网、生物、节能环保、高端装备制造、新能源、新能源汽车等战略性新兴产业。加快实施智能电网、国家北斗、轨道交通、物联网及云计算等一批示范项目和产业基地建设。继续推进石化、汽车、钢铁等支柱产业改造提升转型升级。建设高世代液晶面板、中传重型装备、中石化油品质量升级、桥林上汽基地等一批重大项目。继续实施“千企升级”计划，创建“两化”深度融合示范企业。着力解决小微企业融资难等问题，促进和扶持小微企业发展。

　　加快发展高端服务业。着力推动总部经济、楼宇经济发展，以主城鼓楼区、玄武区、白下区、下关滨江和河西新城、浦口新城、南部新城为重点，布局建设一批高端商务楼宇，重点引进跨国公司、国内百强企业在南京设立功能性总部。做优做强苏宁、雨润、宏图三胞、联创、三宝科技、金鹰、华泰证券、南京证券、江苏银行、南京银行、紫金投资集团等一批有影响的企业总部。继续推进中国软件名城建设，做大做强软件和信息服务业，确保软件服务外包业务收入增长30%以上。推进区域金融中心建设，建设国际金融城，深化宁台金融合作。推进海港、空港、信息港、铁路港建设，加快打造中国航运（空）与综合枢纽名城。推进国家现代服务业城市试点，加快国家数字出版基地、南京广告产业园等现代服务产业园建设。加快发展文化创意、工业设计等产业。积极发展医疗服务等健康产业。支持苏宁易购、焦点科技等电子商务企业做大做强。加快南京国际博览中心二期建设，积极举办国际性展会。

　　切实增强投资和消费拉动。继续实施一批重大产业项目、基础设施项目、环境建设项目，促进消费与投资良性互动。加大产业有效投入，加大对实体经济的扶持力度，确保完成工业和服务业投入均增长15%以上。落实国家鼓励民间投资新36条，进一步促进民间资本投向城市市政设施、产业发展、商务商贸旅游、环境保护、城镇化建设、民生改善等项目。着力推动商贸旅游等优势服务业升级，办好旅游文化节、购物节、美食节、农业嘉年华等活动，大力推动汽车、文化娱乐、教育、健康、节能等方面热点消费。推进紫金山、玄武湖、夫子庙等景区和汤山、桠溪国际慢城、金牛湖、天生桥等旅游休闲度假区建设。积极建设特色街区，促进夜间经济发展。继续落实国家调控政策，促进房地产稳健发展。

　　（二）着力实施创新驱动战略，促进科技与产业融合发展

　　坚持科技创新在全市发展中的核心地位，积极抢占科技和产业发展的制高点。

　　推进科技创新与产业发展相融合。促进科技创新与产业尤其是新兴产业发展、传统支柱产业提档升级共生共长。实施好重大成果转化、重大专利产业化和产学研项目，新建重大公共技术服务平台、战略性新兴产业创新中心和校企联盟，确逼技成果就地转化率达到46%。扶持行业骨干企业牵头与科研院所及高等院校共建联合创新平台，建立国家电网公司智能电网科研产业基地、创新药物“百家汇”等一批产业技术创新联盟。加快“智慧南京”建设，大力发展智慧产业。在新型显示、生物医药、未来网络等领域，着力形成市场规模优势和技术领先优势，加快建设具有全国影响力的战略性新兴产业高地。深化和完善科技创业投融资体系建设，大力发展科技银行、科技保险和科技小额贷款公司，鼓励金融产品和信贷、融资模式创新。

　　增强企业在科技创新中的主体地位。加大对企业技术创新的支持力度。全市内资大中型工业企业和规模以上高新技术企业实现研发机构建设全覆盖。鼓励支持有实力的企业牵头承担国家重点工程。实施市级重点技术创新项目2000项。实施培育高新技术后备企业的“造林工程”，建立500家高新技术后备企业库。积极鼓励科技创业，全面落实科技创业创新政策。继续实施“321人才”计划，引进和培养一批领军型科技创业人才、科技创业家。加强对人才在科技研发、成果转化转让、信息咨询等活动中的收益保护，加大知识产权保护力度，促进小型科技企业成长。

　　加快建设科技创新载体。着力推进国家级高新园区、麒麟生态科技园、软件谷、无线谷、液晶谷、生物医药谷、生物农业谷和仙林科技城、模范马路创新街区、紫金科技创业特别社区等一批重大科技创新载体建设，加快培育在孵企业、高新技术企业、创新型企业。建设国际企业研发园，引进高端研发机构，培育国际联合研究中心。鼓励高等院校、科研院所和企业建立科研设施开放共享运行机制。

　　（三）着力保障与改善民生，提高公共服务能力和水平

　　把人民群众的安危冷暖时刻放在心上，将改善民生、建设幸福都市作为政府工作的着力点。

　　提高城乡居民收入和社会保障水平。采取扎实措施推进城乡居民收入“双倍增计划”，突出普通企业职工、中低收入者和困难家庭增收，统筹提高工资性、经营性、财产性和转移性收入。扶持农民以创业带动就业，多策并举促进农民持续增收。实施更加积极的就业政策，重点解决好高校毕业生、农村转移劳动力、城镇就业困难人员就业问题，全年新增城镇就业14万人。培育扶持自主创业者1万人，创业带动就业7万人。推进和完善社会保险市级统筹，加强社会保险扩面征缴，确保全年社会保险五项险种累计净增参保人员45万人次，城乡基本养老保险、医疗保险和失业保险覆盖率稳定在98%以上。调整城乡低保标准，有条件的区县实现城乡低保同标。

　　切实提供更好的公共服务。推进教育内涵发展，扩大优质教育覆盖面，推动农村优质教育普惠发展，保障外来务工人员子女接受义务教育的权利，加大学前教育投入，提升教育国际化水平，让城乡孩子们接受更好的教育。深化医药卫生体制改革，全面开展医疗救助与基本医保同步结算。仙林国际医院建成运营，加快建设河西新城、浦口新城、南部新城、麒麟新城医疗中心和市公共卫生服务中心。加强院前急救工作。推动文化演艺事业发展，不断改善城乡公共文化设施，实现社区和村文化共享工程服务点全覆盖，进一步丰富人民群众精神文化生活。完成六朝博物馆、江宁织造府、南京书画院、金陵美术馆、城南记忆馆、方志馆工程，加快推进大报恩寺、明皇宫、牛首山、清凉山-石头城等遗址公园，以及科举博物馆、南朝石刻博物馆等重大文化项目的规划建设。完成明城墙玄武门至神策门段环境整治、中山门至琵琶湖段墙体维修—展民国建筑保护与整理修缮工作。

　　加强和创新社会管理。加强信访信息化建设，强化司法保障，完善人民调解、行政调解、司法调解联动的工作体系。注重治安源头管控，着力完善社会治安防控体系，继续推进科技强警，加大违法犯罪打击力度，发展治安志愿者等群防群治组织，夯实平安基层基础，提升群众安全感。完善基层社会管理，引导群众参与社会管理。加强社区自治，推行网格化管理，为社区干部减负，让他们多进门入户服务群众。弘扬南京城市精神，进一步提高市民道德素质。强化基层基础安全生产、消防管理，建立健全药品和食品安全社会管理机制。切实做好双拥、民族宗教和侨务等工作。

　　着重推进民生实事项目。今年的重点是：

　　1、危旧房和城中村改造。改造危旧房65万平方米、城中村190万平方米，逐步改造国有矿区、林场等地棚户区，切实改善群众居住环境。

　　2、改善老旧小区。出新小区70个，整治房屋850幢。完成286个老旧小区二次供水设施改造。实施100部老旧高层住宅电梯整治。

　　3、建设保障房。积极推进中低价商品房、公租房建设，逐步解决“夹心层”的住房问题。新开工保障房300万平方米，竣工500万平方米。

　　4、养老服务。扩建祖堂山养老院，逐步为五保户、低保户等群体提供养老服务。多元投资建设银发社区。

　　5、医疗卫生。在方便市民就医、提高医疗服务技术水平、推进健康管理和因病致贫者及残疾人康复等方面提供更多的惠民服务。

　　6、教育惠民。引入市区名牌中小学进四大保障房片区办学，建成15所中小学、16所幼儿园，确保今年9月份开学。

　　7、农贸市场升级改造。对45个大棚农贸市场进行改造，解决设施老化、环境脏乱差等问题。

　　8、绿道建设。启动100条林荫大道提升、100公里绿色步道建设，推进慢行系统、自行车道、小游园等绿道建设。

　　9、区域供水工程。完成高淳、溧水、栖霞靖安、六合北部、浦口星甸等区域供水工程。

　　10、公交都市建设。南京站综合换乘中心（小红山客运站）投入使用，推进马群换乘中心建设，新增改造公交场站17个，更新公交车1000辆，新增公交专用道20公里、停车位6万个。

　　（四）着力提升城市功能品质，改善市民生活质量

　　城市是我们共同生活的家园，城市的功能品质就是全体市民的生活品质。今年城市建设要更加关注百姓需求，聚焦功能建设、品质提升、环境改善和民生服务。

　　基本完成十项城建节点任务。基本完成青奥板块的主体工程。基本完成街巷整治。基本完成岱山、丁家庄、花岗、上坊四大组团保障房片区建设及功能配套。基本完成城东南片区、城西片区快速路网改造工程。基本完成明外郭-秦淮新河百里风光带上元门到沧波门、秦淮新河段景观建设。基本完成105公里长江干堤提升工程。基本完成主城区的垃圾分类处理。基本完成区域供水全覆盖工程。基本完成村村通公交，全面完成村庄整治任务。基本完成绕城公路沿线江南段和江北段宕口整治。

　　推进基础设施建设。重点抓好轨道交通、过江交通、干线交通建设，地铁3号线、6号线、10号线、11号线实现洞通，推进4号线、宁和、宁高、宁溧轨道交通和河西、麒麟生态科技园有轨电车等工程建设。继续推进纬三路过江隧道建设。稳步推进禄口机场二期、六合机场建设和土山机场搬迁。推进龙潭国际综合物流集聚区及西坝、滨江、七坝三大港口物流节点建设。宁杭城际铁路通车，推进宁安城际铁路建设和宁芜铁路改造，做好沪宁城际铁路站房建设。加快绕城公路和纬一路快速化改造，推进宁高新通道、江北大道、石扬路、光华路东延等建设改造。

　　加大功能性项目建设力度。进一步优化城市布局、城市功能，完善城乡规划、城市设计。加快推进苏宁慧谷、金鹰三期、中城国际广场等功能性项目建设，积极推进河西中央商务区二期和海峡城、美国富顿世贸中心等建设，打造一批承载功能强的总部经济载体。规划建设一批国际化生活社区，营造国际化生活氛围。建设浦口新城江滩湿地公园、雅居乐等项目。推进南部新城南京南站南广场开发建设，加快大明路、宁南汽贸区企业搬迁。推进麒麟生态科技园科技综合体、基础设施和生态环境建设。加快下关滨江航运服务中心等商务楼宇建设，推进大马路地区民国建筑的保护维修、滨江岸线风光带建设。整修改造老城南地区门东历史街区，并逐步对外开放。加快实施江心洲生态科技岛新纬壹科技城等工程。继续推进跨江发展，积极争取将“江北新区”上升为国家区域战略。推进“宁镇扬”同城化、南京都市圈发展。

　　提高城市管理水平。实施《南京市城市治理条例》，推进城市管理向城市治理转变，让人民群众更多地参与城市建设和管理—展以“平安和谐、交通秩序、市容市貌、卫生安全、绿化美化”等为主要内容的环境整治。重点推进河西地区、新街口-鼓楼地区、紫金山-玄武湖地区、城市出入口地区综合整治，完成17条主干道整治任务。建成江南、江北静脉产业园一期，在全市推广垃圾分类，建设垃圾清运资源回收处理系统。落实区、街管理城市的责任，整体提升城市管理规范化、精细化水平。

　　改造危旧房和城中村，改善市民居住质量，让市民生活得更有尊严是今明两年的攻坚任务。今年重点实施应天高架桥沿线、河北村、清河村、南河沿线、二桥南沿线和迈皋桥地区等城中村、危旧房搬迁计划。

　　（五）着力加快城乡一体化建设，切实提升全域统筹发展水平

　　建设富饶的美丽乡村，是城乡人民共同的愿景，需要我们下大力气推进。

　　加快先进制造业向郊区县集聚。培育科技含量高、核心竞争力强的骨干企业，加快形成主导功能明确、产业特色突出的郊县先进制造业发展新格局。全力建设宁高、宁溧科技园区，推进高淳、溧水两个千亿级制造业基地建设。着力推进高新区向自主创新示范区、化工园向循环型生态工业园区转变，加快推进经济技术开发区、江宁开发区向高端制造业基地、新兴产业基地、创新创业示范基地转型，加快形成以国家级开发区为龙头、省级开发区和专业园区优势互补的郊县产业发展格局。

　　加快农业现代化进程。重点做好土地综合整治工作，盘活农村土地等资源。推进农业“1115工程”，发展现代农业，新建高标准农田14万亩，新增设施农业8万亩，新培育10个市级现代农业示范园区，促进人才、科技、资金等要素向现代农业园区集中。加快休闲农业发展，办成农村富民产业。大力发展农民专业合作组织，转变农业生产经营机制，新增农民专业合作社、土地股份合作社400家。加快滁河、水阳江防洪治理工程建设，完成农田水利连片治理28万亩。

　　综合推进城镇化和美丽乡村建设。按全市城镇体系规划有序推进新市镇、新社区建设，统筹兼顾经济发展、产业布局、生态环境、城市功能，统筹安排户籍、土地、住房、社保、就业、公共服务。抓住城镇化发展新机遇，加快建设永阳、淳溪、雄州等新城，做好谷里、竹镇等10个试点街镇建设，完善新市镇、新社区生活居住、公共服务配套等功能，挖掘和保护农村历史文化遗存、乡土风俗、村庄特色建筑等。规划江宁谷里-横溪、高淳桠溪-漆桥、溧水白马-无想山、浦口汤泉、六合竹镇-金牛湖等五个片区1000平方公里的美丽乡村示范区。建设十大特色旅游村镇，打造乡村旅游精品线路—展新一轮经济欠发达村镇帮促工作，加强村级能力建设。继续推进郊县公路网建设，完成干线公路建设150公里。

　　（六）着力加强环境保护，进一步加快生态文明建设

　　给子孙后代留下“天蓝、地绿、水净”的美好家园，加强生态文明建设，需要政府攻坚克难，持续不断地努力。

　　实施环境综合治理。重点治理燃煤锅炉、工地扬尘、道路扬尘、汽车尾气、秸秆焚烧等影响大气环境的顽症，以及噪音、油烟扰民等群众反映强烈的环境问题，力争空气优良天数比例达到88％以上。重点推进城北、桥北、铁北等污水处理厂扩能升级改造和配套管网建设，建设一批街镇污水处理厂。着力推进河西地区水环境整治，实施龙江和南湖片区雨污分流改造工程，完成南十里长沟主流河道，以及幸福河、南河、清江河、龙江河等20条以上黑臭河道整治任务，继续实施街巷、工企单位、商业楼宇雨污分流管网建设。

　　加大生态建设与保护力度。加快推进生态文明建设，围绕创建国家生态市、国家生态园林城市、国家森林城市，深入推进绿色南京建设，加快建设美丽南京。加强生态保护区建设，完善绿化评估、绿线控制等制度。重点做好紫金山-玄武湖中央公园、明外郭-秦淮新河百里风光带和滨江两岸风光带建设，推进青龙山、老山、牛首山、幕府山和栖霞山五大郊野公园建设，实施青奥会重点区域园林绿化提升工程，整治主干道绿化，加强绿荫道建设。新增20个小游园，完成40个绿色人居环境社区创建和提升工作，绿化村庄500个。加强生态修复，做好采石宕口治理和复绿工作。新增造林7万亩，森林覆盖率提高到28%。规划启动石臼湖、金牛湖两大备用水源地建设，确保饮用水源地水质达标率为100%。

　　全力推进节能减排。继续推动主城区工业企业向开发园区转移。严格控制排污总量，继续淘汰落后产能、整治“三高两低”企业，完成金陵石化催化裂化装置烟气脱硫改造，完成华润热电、化工园热电、华能南京及扬子、南化等燃煤锅炉烟气脱硝工程。从严控制新增燃煤量项目，严格控制高耗能、高排放和产能过剩行业增长—展清洁生产、循环综合利用，重点推进节能改造和循环经济发展项目。完善工业污水处理系统，全面推进大型工业企业污水处理设施升级改造建设。

　　（七）着力推进重点领域改革，实施更加积极主动的开放战略

　　坚持社会主义市场经济的改革方向，坚持对外开放的基本国策，推进关键领域改革，让社会充满活力。

　　深化各项综合改革。深入落实《关于实施综合改革工程的意见》。深化科技体制改革，引导科技资源向社会开放，注重科技创新质量和科技创业成长率。推进市区财政体制改革，聚焦重点，集中力量办大事，加大对重点领域和薄弱环节的支持力度，把有限的资金用在刀刃上。实施10个政府部门预算信息公开。按照国家和省的部署，推动收入分配制度改革。扎实推进营业税改增值税试点工作。推进公立医院管理体制改革，实施公费医疗改革。继续深化转变土地利用方式改革创新。深化投融资体制改革，促进投融资良性运行。推进城市管理体制改革，实现道路基础设施维护、清扫保洁、绿化养护市场化。深化公共管理创新，健全政府购买公共服务机制，鼓励发展社会中介服务组织。

　　提高对外开放的层次。加快城市国际化进程，着力加强城市国际化创新、商务、文化、会展旅游、人居等功能，积极参与国际分工、国际循环、国际竞争。拓展国际交流合作，推动引资、引技、引智有机结合，强化对美、德、英、法、日、韩等国家招商，加强宁台、宁新项目合作，突出新兴产业和高端服务业等产业招商，引进一批代表国际先进水平、对产业升级有重大带动作用的外资大项目。积极推动跨国公司来宁设立地区总部、投资性公司、采购和结算中心等外资功能性机构。进一步推进省级开发区权力下放工作，加快园区转型发展，继续推进开发园区“扩容”工作。南京综合保税区封关运作，金陵海关全面运行。实施“科技兴贸”战略，培育特色产业链和创新集聚区，扩大高附加值产品出口，培育外贸出口新增长点。大力发展服务贸易。帮助企业开拓国际市场、利用海外资源，增强企业国际化经营能力，努力把南京本土企业培育成国际化知名企业。

　　各位代表，今年将举办亚青会，这对明年青奥会的成功举办至关重要，全市上下要聚焦重点，高标准办好这届体育盛会。加快青奥村、国际风情街、青奥轴线、青奥体育公园等工程建设，完成亚青会运动员村及其场馆维修改造，确保达到赛事功能要求。践行青奥特色理念，突出中国文化和南京元素，策划开展各类文化教育活动。深入实施全民健身计划，加快建设亚洲体育中心城市和世界体育名城。切实做好城市运行保障等工作，确保亚青会成功举办，提升南京城市国际形象。

　　（八）着力强化依法行政，努力建设人民满意的法治型、服务型政府

　　依法行政是政府履行职责的基本准绳，转变作风是政府为民服务的重要保障。

　　扎实转变工作作风。人民政府要与人民群众心心相印，坚持为民掌权、为民谋利、为民办事、为民服务。要真正到群众中去倾听民声，了解民情，顺应民意，科学决策。实干兴邦，空谈误事，大力倡导敢于负责、勇于担当、言必行、行必果的优良风气，切实改进文风会风，精简会议活动，精简文件简报。不断提升"12345"热线服务水平，继续开展“政风行风热线”媒体联动和“向人民汇报”民主评议直播活动。厉行勤俭节约，加强廉洁从政，依法严惩以权谋私、失职渎职等违法违纪行为。着力整治不作为、不出力、不落实的不良行为，提高政府行政团队的执行力和公信力。

　　切实加强依法行政。政府要坚持依法行政、依法管理、依法办事，加快推进法治政府建设，着力规范政府行为，加强重点领域执法监督。增强全民法制观念和守法意识。拓宽公众参与立法渠道，提高行政立法质量。健全规范性文件征求意见制度。加大对公共资源交易的监管力度，务实推行阳光拆迁，全面实施房屋拆迁补偿公示，着力解决损害群众利益的突出问题。深化行政审批制度改革，积极推进市、区县政务服务中心的对接，确保许可审批集中办理、一网运行。认真开展“十二五”规划中期评估。切实做好全国第三次经济普查工作。

　　广泛接受各界监督。对政府部门实施绩效管理，加强行政督察和审计监督。落实服务承诺制，切实增强行政执行力。自觉接受人大及其常委会的法律监督与工作监督，积极支持人民政协履行政治协商、民主监督、参政议政职能，切实做好人大代表建议、批评和意见及政协提案办理工作。广泛听取各民主党派、工商联、无党派人士及各人民团体的意见和建议，高度重视司法监督、舆论监督和社会公众监督。

　　各位代表，新的征途已经开启，美好的未来召唤着我们，新一届政府责任重大。让我们紧密团结在以习近平同志为总书记的党中央周围，以科学发展观统领经济社会发展全局，为南京城市的发展，为南京人民的福祉，凝心聚力地干，求真务实地干，团结拼搏地干，为率先基本实现现代化而努力奋斗！
